# Advanced mare age impairs the ability of in vitro‐matured oocytes to correctly align chromosomes on the metaphase plate

**DOI:** 10.1111/evj.12995

**Published:** 2018-08-09

**Authors:** M. Rizzo, K. D. Ducheyne, C. Deelen, M. Beitsma, S. Cristarella, M. Quartuccio, T. A. E. Stout, M. de Ruijter‐Villani

**Affiliations:** ^1^ Department of Equine Sciences Faculty of Veterinary Medicine Utrecht University Utrecht the Netherlands; ^2^ Department of Veterinary Sciences Messina University Messina Italy; ^3^ Department of Veterinary Medical Imaging and Small Animal Orthopaedics Faculty of Veterinary Medicine Ghent University Merelbeke Belgium

**Keywords:** horse, oocytes, maternal ageing, spindle morphology, chromosome misalignment

## Abstract

**Background:**

Advanced mare age is associated with declining fertility and an increased risk of early pregnancy loss. Compromised oocyte quality is probably the primary reason for reduced fertility, but the defects predisposing to embryonic death are unknown. In women, advanced age predisposes to chromosome segregation errors during meiosis, which lead to embryonic aneuploidy and a heightened risk of miscarriage.

**Objectives:**

To evaluate the effect of advanced mare age on chromosome alignment and meiotic spindle morphology in in vitro‐matured (IVM) oocytes.

**Study design:**

Morphometric and morphological analysis.

**Methods:**

To investigate differences in spindle organisation and chromosome alignment between young and old mares, oocytes collected from slaughtered mares were divided into two groups depending on mare age (young, ≤14 years and old, ≥16 years), IVM and stained to visualise chromatin and alpha‐tubulin. Spindle morphology, morphometry and chromosome (mis)alignment were evaluated by confocal microscopy and 3D image analysis.

**Results:**

Oocytes from old mares showed a higher incidence of chromosome misalignment (47.4% vs. 4.5%; P<0.001) and a thicker metaphase plate (mean ± s.d.: 5.8 ± 1.0 μm vs. 4.9 ± 0.9 μm; P = 0.04) than oocytes from young mares. Although no differences in spindle morphometry were detected between old and young mares, an increased major spindle axis length was associated with chromosome misalignment (mean ± s.d.: 25.3 ± 6.1 μm vs. 20.8 ± 3.3 μm; P = 0.01) irrespective of age.

**Main limitations:**

The oocytes were IVM and may not exactly reflect chromosome misalignment in vivo.

**Conclusions:**

Advanced mare age predisposes to chromosome misalignment on the metaphase II spindle of IVM oocytes. The compromised ability to correctly align chromosomes presumably predisposes to aneuploidy in resulting embryos and thereby contributes to the age‐related decline in fertility and increased incidence of early pregnancy loss.

**The Summary is available in Portuguese – see Supporting Information**

## Introduction

Advanced mare age (in particular beyond 14 years) is associated with a decline in fertility 1, 2, 3. This manifests as a lower success of fertilisation in older (81%) than younger mares (96%) 4, and a trebling of the incidence of pregnancy loss between Day 12 and Day 60 in mares aged 18 years or older (30%) compared with mares younger than 12 years (10%) 5, 6, 7, 8. Estimated pre‐pregnancy detection losses (between Day 2 and Day 14 after ovulation) are also significantly higher in old (62%; 19.4 ± 1.0 years) than in young mares (9%; 5.7 ± 0.3 years) 4. The cause(s) of this decline in fertility and associated increase in the incidence of pregnancy loss are likely to be multifactorial; nevertheless, intrinsic oocyte defects are thought to be a major contributor, because transferring oocytes from old mares into the oviduct of younger recipients does not improve the likelihood of fertilisation or reduce the risk of pregnancy loss 5.

During development, oocytes undergo two rounds of chromosome segregation (Meiosis I and Meiosis II) to produce a haploid gamete competent to develop into a diploid zygote and embryo, after fusion with the paternal haploid set of chromosomes introduced by the spermatozoon during fertilisation. During oocyte meiosis, the formation of Metaphase I (MI) and Metaphase II (MII) spindles and accurate alignment of chromosome pairs (homologous chromosomes in MI; sister chromatids in MII) on either side of the equatorial plate are required for the correct segregation of the chromosomes between the oocyte and the polar bodies, to ensure genomic integrity. In women, advanced age predisposes to chromosome segregation errors in oocytes during both meiotic divisions, which in turn leads to an increased incidence of embryonic aneuploidy, early miscarriage and birth defects such as Down syndrome 9, 10. In particular, advanced maternal age seems to impair the ability of the oocyte to detect and correct the presence of misaligned chromosomes 10. While in vitro maturation has been shown to compromise the process of chromosome segregation in mares, the effect of maternal age on the ability to correctly form a spindle and align the chromosomes is unknown 11. The purpose of this study was to determine whether advanced mare age affects spindle morphology and interferes with proper alignment of chromosomes in in vitro‐matured MII oocytes.

## Materials and methods

### Oocyte collection and culture

Ovaries were recovered from slaughtered mares within 15 min of death and divided into two groups, depending on the age of the mare (young, ≤14 years and old, ≥16 years) as determined by examining the microchip number and associated passport. Ovaries were packaged at 21–25°C as previously described for short shipment 12 and transported to the laboratory within 5 h. Cumulus–oocyte complexes (COCs) were then immediately recovered from the ovaries by scraping the follicle wall with a bone curette and flushing the dislodged cells with embryo flushing medium (Euroflush)^a^, as described by Hinrichs *et al*. 13. Only follicles ≤30 mm were selected for scraping, to avoid the collection of oocytes that had already matured. Recovered COCs were evaluated with a dissecting microscope at 10–60× magnification, and only oocytes with at least one layer of intact cumulus cells were used, irrespective of the morphology of the cumulus (expanded or compact); denuded oocytes were discarded. COCs were then matured in a 50:50 mixture of Dulbecco's minimal essential medium (DMEM) and Ham's F12^b^ supplemented with 10% fetal calf serum^c^, 0.125 μg/mL epidermal growth factor^d^, 0.1 IU/mL follicle‐stimulating hormone^c^, 0.6 mmol/L cysteine^c^, 0.1 mmol/L cysteamine^c^, 0.1% insulin^e^, 0.1% transferrin^e^ and 0.1% sodium selenite^e^ for 26 h at 38.5°C in a humidified atmosphere of 5% CO_2_ in air. After maturation, cumulus cells were removed by brief exposure to Hepes‐buffered synthetic oviduct fluid (H‐SOF)^f^ containing 1 μg/mL of hyaluronidase^c^ followed by gentle pipetting through a fine bore pipette. Only oocytes displaying a first polar body were used for further analysis.

### Oocyte fixation and immunostaining

Oocytes were fixed in microtubule‐stabilising solution for 1 h at 38°C (medium M^14^), followed by 2% paraformaldehyde for 30 min at room temperature and stored in phosphate‐buffered saline (PBS)^g^ at 4°C until further processing. Prior to staining, the oocytes were washed three times in PBS containing 3 mg/mL polyvinylpyrrolidone^c^ (PBS‐PVP) for 5 min. Next, they were incubated overnight at 4°C in PBS containing a 1:250 dilution of a mouse monoclonal anti‐α‐tubulin antibody (T5168)^c^, to label the microtubules in the spindle. The oocytes were then washed twice in 0.1% bovine serum albumin (BSA)^c^ in PBS for 5 min and incubated for 1 h at room temperature in a blocking solution containing 0.1 mol/L glycine^c^, 1% goat serum^c^, 0.01% Triton X‐100^c^, 0.5% BSA^c^ and 0.02% sodium azide^c^ in PBS. Thereafter, the oocytes were incubated for 1 h at 37°C in PBS containing 0.5% Triton X‐100, 0.5% BSA and 1:100 goat anti‐mouse Alexa Fluor^®^ 488 antibody (A11029)^h^ Following two 5 min washing steps in PBS supplemented with 0.1% BSA and 0.1% Triton X‐100 and one in PBS alone, the oocytes were incubated for 30 min at room temperature in PBS‐PVP containing 1:100 Hoechst 33342^c^ to label the DNA, washed in PBS‐PVP for 5 min and mounted on glass slides^i^ with anti‐fade mounting medium^j^.

### Image acquisition and analysis

**Figure 1:**
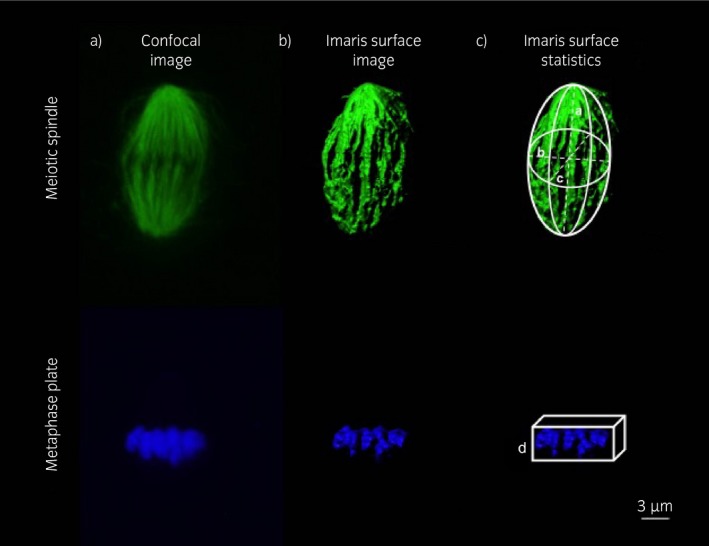
Example of surface rendering of the meiotic spindle and metaphase plate in vitro*‐*matured mare oocytes. a) Confocal image prior to processing; b) image after processing with the Imaris surface tool; c) illustration of the measurements used for the statistics (a, pole‐to‐pole length; b and c, spindle width; d, metaphase plate thickness). The scale bar is set at 3 μm.

Image acquisition was performed using a Leica TCS‐SP5 confocal laser scanning microscope equipped with a 63× objective^k^ . Hoechst 33342 was stimulated with a 351‐nm laser, and emission was detected between 414 and 466 nm (blue channel), Alexa Fluor 488 was separately stimulated with a 488‐nm laser, and emission was detected in the 511–577 nm range (green channel). The microtubular spindle, labelled with Alexa Fluor 488, and the chromosomes, labelled with Hoechst 33342, were identified using sequential confocal sections (Z‐stacks) at 0.42‐μm intervals. A three‐dimensional (3D) image of the spindle and chromosomes was then created and analysed using Imaris 8.2 software^l^ . The spindle was assessed for gross morphological parameters (Table 1). The shape of the spindle was classified as ‘normal’ if it was bipolar and fusiform, or ‘abnormal’ if it was tri‐ or tetrapolar or severely misshapen. Chromosomes were considered to be misaligned when they were displaced by ≥2 μm from the metaphase plate, as described by Coticchio *et al*. 15. Chromosome misalignment was only assessed in morphologically normal spindles and was scored as absent if all of the chromosomes were properly aligned along the spindle equator, mild when up to five chromosomes were positioned ≥2 μm from the equator or severe when more than five chromosomes were distant from the equator (Table 1; Supplementary Items [Link], [Link], [Link]). The Imaris surface tool was used to render solid surfaces best representing both the spindle and the chromosomes, regardless of spindle orientation (Supplementary Item 4). To create a 3D image of the meiotic spindle, the green channel was selected, a Gaussian smoothing filter was applied (detail level of 0.0853 μm), and a threshold for surface creation was selected on the basis of absolute intensity using 51 and 166 arbitrary units as lower and upper thresholds, respectively. The 3D image for the metaphase plate was created in a similar fashion by selecting the blue channel and using 27.2 and 213 arbitrary units, respectively, as the lower and upper thresholds for absolute fluorescence intensity. If the polar body was in close proximity to the spindle, this was filtered out on the basis of size and or position (x, y, z). Three‐dimensional measurements of the spindle (pole‐to‐pole length, width and volume) were taken from the ellipsoid statistics (Fig 1). DNA dispersion over the metaphase plate was assessed by creating a three‐dimensional bounding box (a rectangular prism) around the metaphase plate and by measuring the width of this box (the length of the side parallel to the spindle's main axis) (Fig 1). Misaligned chromosomes were not included in the bounding box (Supplementary Item 4).

**Table 1:** Diagram illustrating the classification of in vitro*‐*matured mare oocytes into normal and abnormal Metaphase II spindle shapes and by chromosome alignment

Spindle type	Definition	Diagrammatic representation
Normal without misalignment	Bipolar fusiform shape with all chromosomes aligned at the equator	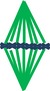
Normal with mild misalignment	Bipolar fusiform shape with one to five chromosomes displaced from the equator	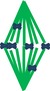
Normal with severe misalignment	Bipolar fusiform shape with more than five chromosomes displaced from the equator	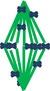
Tri‐ or tetrapolar	Three or four defined poles	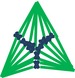
Severely misshapen	Poorly defined or missing poles	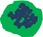

### Data analysis

Statistical analysis was performed using Statistical Package for the Social Sciences (SPSS) 24.0 software^m^. To analyse differences between groups, Fisher's exact test was used for categorical variables (spindle type and chromosome misalignment), and independent *t* tests were used to compare spindle and metaphase plate morphometric parameters. Significance was set at P≤0.05.

## Results

A total of 303 COCs were recovered from 51 mares; 169 COCs from 27 young and 134 COCs from 24 old mares. Mare ages ranged between 2 and 14 years (mean ± s.d.: 7.5 ± 3.5 years) for the young group and between 16 and 27 years (mean ± s.d.: 19 ± 3.9 years) for the old group. No significant difference (P = 0.5) between the two groups was observed for the success of first polar body extrusion after maturation, with 75 of the 169 (44.4%) oocytes from young mares and 54 of the 134 (40.3%) oocytes from old mares reaching MII. Per group, 25 MII oocytes were randomly selected to analyse spindle morphology and chromosome alignment. Of these, three oocytes from the young group and six from the old group had to be excluded from the final analysis as a result of insufficient immunofluorescent staining of the chromatin.

### Advanced mare age compromises the ability of in vitro‐matured MII oocytes to align chromosomes

**Figure 2:**
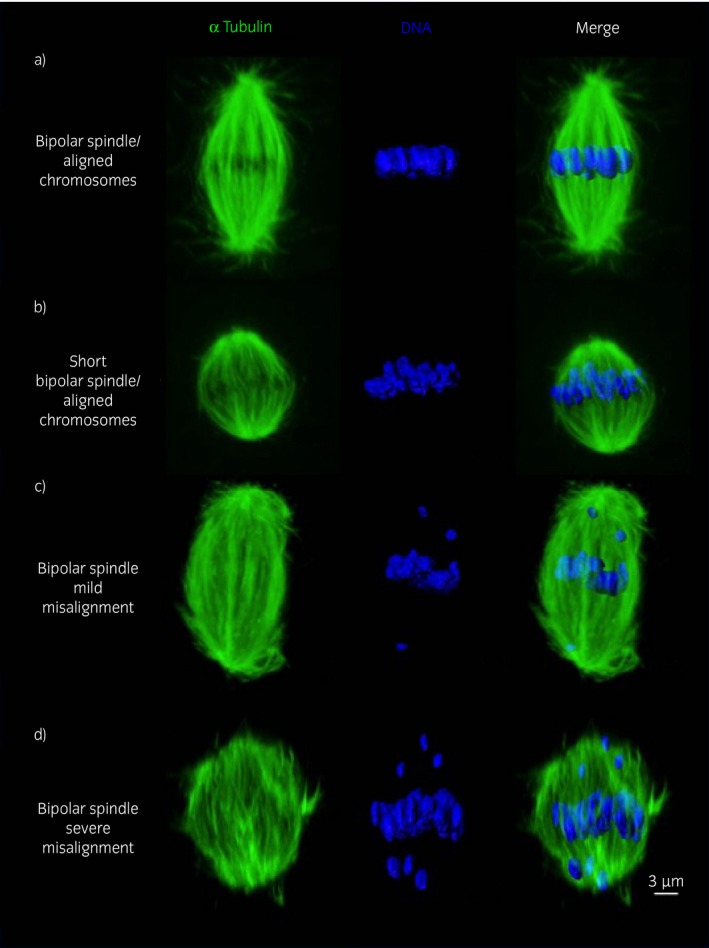
Representative images of a) a bipolar spindle with properly aligned chromosomes on the metaphase plate, from a young mare oocyte; b) bipolar spindle showing a reduced pole‐to‐pole length but with chromosomes properly aligned on the metaphase plate, from an old mare oocyte; c) bipolar spindle with increased length showing mild chromosome misalignment (≤5 displaced chromosomes), from an old mare oocyte; d) bipolar spindle showing severe chromosome misalignment (>5 displaced chromosomes), from an old mare oocyte. The scale bar is set at 3 μm.

**Figure 3:**
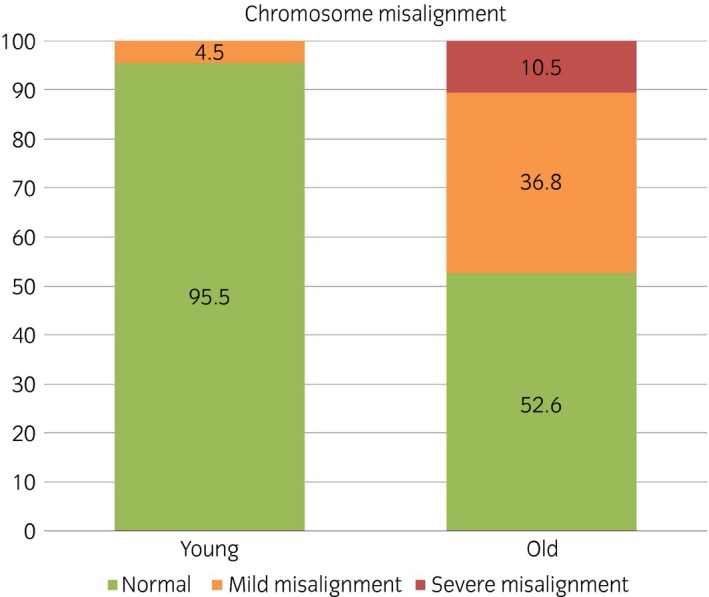
Frequency of chromosome misalignment in MII oocytes from young (≤14 years) and old (≥16 years) mare. Absent: all chromosomes aligned on metaphase plate; mild: 1–5 misaligned chromosomes; severe: >5 misaligned chromosomes.

Representative images of spindles and metaphase plates are shown in Fig 2. MII oocytes from old mares showed a significantly higher incidence of chromosome misalignment (47%) compared with oocytes from young mares (5%) (Fig 3; P<0.001). Moreover, while misalignment in the young group was limited to a single oocyte with a single chromosome displaced from the equator, in the old group both mild misalignment (37%) and severe misalignment (11%) were detected.

### MII oocytes from old mares have a thickened metaphase plate

**Figure 4:**
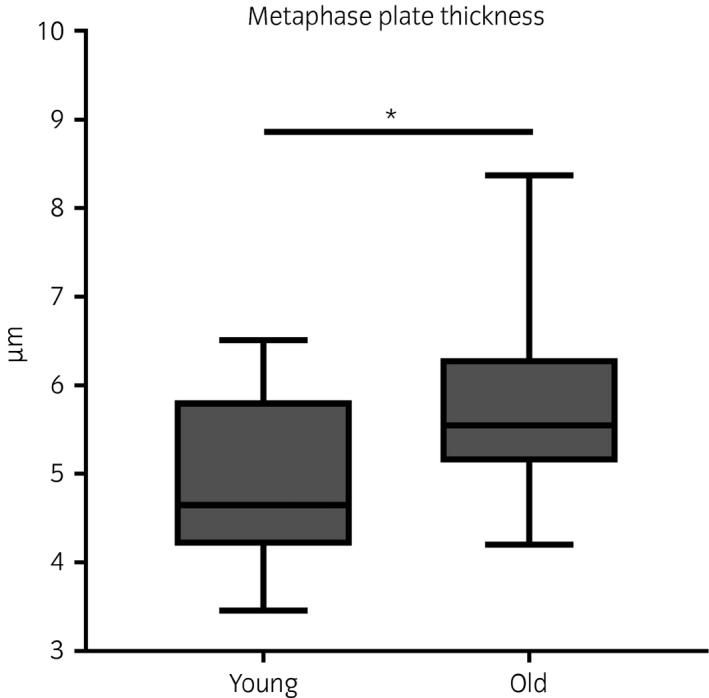
Box plot representation of metaphase plate thickness in the oocytes of mares of different ages (young: ≤14 years and old: ≥16 years). Asterisks indicate significant differences, P<0.05.

DNA dispersion over the metaphase spindle was increased in oocytes from old mares, which showed a significantly (P = 0.004) thicker metaphase plate (mean ± s.d.: 5.8 ± 1.0 μm) than young mares (4.9 ± 0.9 μm) (Fig 4).

### Spindle length is increased in oocytes displaying chromosome misalignment

**Figure 5:**
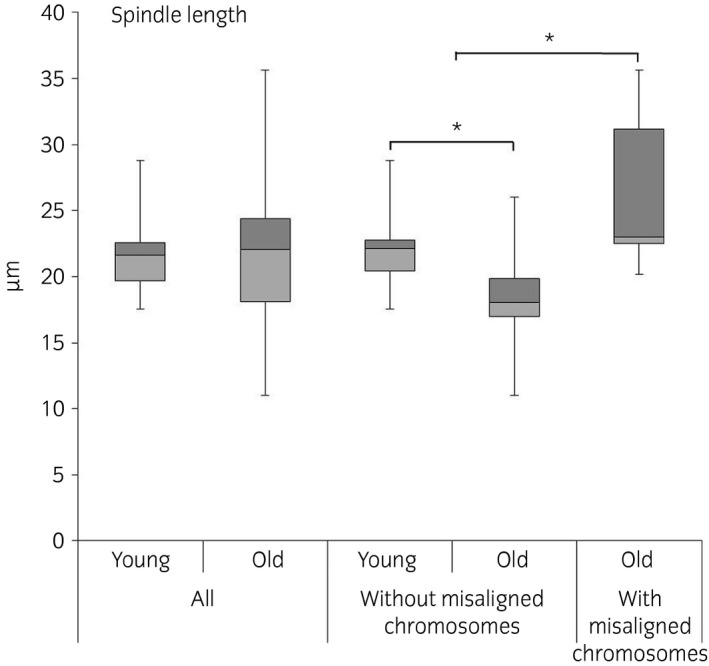
Box plot representation of spindle length (pole‐to‐pole) in the oocytes of mares of different ages (young: ≤14 years and old: ≥16 years) and morphological (aligned vs. misaligned chromosomes) classes. Asterisks indicate significant differences, P<0.05.

All oocytes from young mares and all but one of the oocytes from old mares showed morphologically normal bipolar spindles. The one oocyte from an old mare with a grossly abnormal spindle displayed a tripolar spindle (Supplementary Item 5). Using the 3D image analysis, it was possible to examine spindle morphology methodically in detail, and to record parameters such as spindle major axis dimensions (axes a, b and c; Fig 1; Supplementary Item 4). Although no significant differences in spindle morphometric parameters were detected between oocytes from old and young mares (with a mean ± s.d.: length 21.5 ± 2.5 μm (P = 0.7) and width 12.3 ± 1.9 μm (P = 0.5) in young mare oocytes and 22.2 ± 6.0 μm and 11.8 ± 2.9 μm in old mare oocytes, respectively), the length of the major spindle axis was increased in oocytes displaying chromosome misalignment (mean ± s.d.: 25.3 ± 6.1 μm vs. 20.8 ± 3.3 μm; P = 0.05) irrespective of mare age (Fig 5). Consequently, spindle length was highly variable within the old mare group (range 11–35 μm), while it was more consistent in the young mare group (range 17–28 μm). Moreover, spindles without misalignment were smaller in old mare than young mare oocytes. In fact, when oocytes with misaligned chromosomes were not considered, both spindle length (mean ± s.d.: 18.3 ± 4.2 μm vs. 26.6 ± 6.4 μm; P = 0.01) and width (mean ± s.d.: 10.4 ± 2.1 μm vs. 13.4 ± 3.2 μm; P = 0.01) in oocytes from old mares were significantly reduced compared with young mares (Fig 5; Supplementary Items 1 and 6).

## Discussion

In vitro‐matured MII oocytes from old mares showed a higher incidence of chromosome misalignment than oocytes from young mares 16. Similarly in women, Battaglia *et al*. 9 reported that 79% of the MII spindles in oocytes from 40‐ to 45‐year‐old women showed clear displacement of chromosomes from the metaphase plate, whereas only 17% of MII spindles in oocytes from 20‐ to 25‐year‐old women showed chromosome misalignment; they proposed that age may alter spindle components and/or the timing of the meiotic phases and thereby predispose to irregularities of the microtubular spindle and chromosome misalignment. As defects in chromosome alignment have been shown to be predictive of aneuploidy in oocytes 9, it is probable that the increased incidence in misalignment detected in MII oocytes in mares >16 years old will be reflected by an increased prevalence of aneuploidy in fertilised oocytes, zygotes and embryos from older mares. The mechanism underlying the increased incidence of chromosome misalignment in oocytes from aged females is unknown; however, Yun *et al*. 17 suggested that dysfunction of the spindle assembly checkpoint (SAC), a mechanism involved in regulating and correcting kinetochore–microtubule attachment prior to anaphase, might play an important role in this process. Yun *et al*. 17 found that the SAC in oocytes from aged mice was more ‘permissive’, that is allowed anaphase to progress before chromosome alignment at the metaphase plate was complete, and unable to correct ‘faulty’ attachment between the kinetochores and the microtubules of the spindle; in particular, some SAC components such as mitotic arrest deficient 2 (MAD2) and aurora kinase C (AURKC) showed reduced function, especially when the oocytes were matured and handled in vitro 17. We propose that compromised function of the SAC may also contribute to impaired chromosome alignment and segregation in aged mares and increase the risk of aneuploidy in mature oocytes.

Morphometric analysis revealed that the meiotic spindle in horse oocytes has axial dimensions of approximately 21 × 12 μm. These measurements differ from those previously reported by Franciosi *et al*. 11 (19.89 ± 1.15 μm vs. 17.28 ± 0.81 μm); however, the latter authors measured the spindle axes on the basis of a projected image rather than using confocal microscopic Z‐stacks and 3D image reconstruction and analysis, as in the present study. Our results show that the spindle in horse oocytes is larger than that in human oocytes (12 × 9 μm) 15 but smaller than in mice oocytes, where the major axis ranges from 20 to 30 μm 18. In addition, the length of the MII spindle in horse oocytes seems to be influenced by both maternal age and chromosome misalignment. In fact, when spindles with misaligned chromosomes were not considered, advanced mare age was associated with a significant reduction in pole‐to‐pole spindle length. This is similar to what has previously been reported for aged, inbred (CBA) mice 19 and may result from compromised activity of microtubule motor proteins in oocytes from aged females. There was also a relationship between chromosome alignment and spindle length in horse oocytes, with spindle length being significantly longer in oocytes with misaligned chromosomes. Bromfield *et al*. 20 previously reported a relationship between the number of displaced chromosomes and the overall length of the meiotic spindle in frozen–thawed human oocytes.

The percentage of slaughterhouse‐derived oocytes that successfully matured to the MII stage in the present study is similar to that reported in previous studies (20–85%) 21, 22, and the absence of a significant difference in the percentage of oocytes reaching MII between young and old mares mirrors the findings of Brinkso *et al*. 23 who reported similar development to MII for young (≤15 years; 28/168) and old mares (>15 years; 11/66). However, meiotic spindles require considerable energy (e.g. GTP and ATP) for assembly and to ensure maintenance of bipolarity and correct interaction between the kinetochores and the microtubules, which in turn ensure correct alignment of the chromosomes onto the metaphase plate. It has previously been shown that oocytes from aged mares are more susceptible to mitochondrial damage during in vitro maturation 24, 25, and it is therefore possible that, during in vitro maturation, oocytes from aged mares are unable to satisfy the energy requirements to ensure spindle integrity and correct microtubule–kinetochore interaction, thereby increasing the risk of improper spindle assembly and chromosome misalignment.

In conclusion, the present study demonstrates that advanced mare age compromises the ability of in vitro‐matured equine oocytes to correctly align their chromosomes along the metaphase plate. This is likely to predispose to aneuploidy in the oocytes after fertilisation‐induced completion of the second meiotic division and in resulting embryos. This may in part explain the age‐related reduction in oocyte developmental competence and the increase in the incidence of early pregnancy loss observed in aged mares.

## Authors’ declaration of interests

No competing interests have been declared.

## Ethical animal research

Research ethics committee oversight is not required by this journal: the study was performed on material obtained from an abattoir.

## Sources of funding

K.D. Ducheyne's salary was funded by the Agentschap voor Innovatie door Wetenschap en Technologie (IWT, Grant 141492).

## Authorship

M. de Ruijter‐Villani and T.A.E. Stout conceived the study. M. Rizzo and K.D. Ducheyne developed the experimental design with advice from M.S. Cristarella, M. Quartuccio, T.A.E. Stout and M. de Ruijter‐Villani. M. Rizzo and K.D. Ducheyne performed the experiments with the support of C. Deelen and M. Beitsma. K.D. Ducheyne, M. Rizzo and M. de Ruijter‐Villani performed interpretation of the data. T.A.E. Stout and M. de Ruijter‐Villani supervised the project. M. de Ruijter‐Villani, K.D. Ducheyne and M. Rizzo wrote the manuscript, which was edited by T.A.E. Stout. All authors have approved the final version of the manuscript.

## Supporting information


**Summary in Portuguese.**

**Supplementary Item 1**: 3D‐video of a normal equine oocyte originating from a young (≤14 years) mare with a bipolar metaphase II spindle without chromosome misalignment.

**Supplementary Item 2:** 3D‐video of an equine oocyte originating from an old (≥16 years) mare showing a bipolar metaphase II spindle with mild chromosome misalignment (i.e. 1–5 misaligned chromosomes).

**Supplementary Item 3:** 3D‐video of an equine oocyte originating from an old (≥16 years) mare showing a bipolar metaphase II spindle with severe chromosome misalignment (i.e. >5 misaligned chromosomes).

**Supplementary Item 4**: Video demonstrating the surface rendering of the metaphase II spindle and metaphase plate of an in vitro*‐*matured mare oocyte using Imaris 8.2 software.

**Supplementary Item 5:** 3D‐video of an equine metaphase II stage oocyte originating from an old (≥16 years) mare showing a tripolar spindle.

**Supplementary Item 6:** 3D‐video of an equine oocyte originating from an old (≥16 years) mare showing a bipolar metaphase II spindle with a shortened pole‐to‐pole length.

## Manufacturers' addresses

^a^IMV Technologies, Leeuwarden, the Netherlands.

^b^GIBCO BRL Life Technologies, Bleiswijk, the Netherlands.

^c^Sigma‐Aldrich Chemical Co., St. Louis, Missouri, USA.

^d^Peprotech Inc., Rocky Hill, New Jersey, USA.

^e^Corning Life Science, New York, USA.

^f^Avantea, Cremona, Italy.

^g^B. Braun, Hessen, Germany.

^h^Invitrogen Corp., Carlsbad, California, USA.

^i^Superfrost Plus, Menzel, Braunschweig, Germany.

^j^Vectashield, Vector Laboratories, Burlingame, California, USA.

^k^Leica Microsystems, Wetzlar, Germany.

^l^Bitplane AG, Zurich, Switzerland.

^m^IBM, Armonk, New York, USA.
