# Supplementary material for: Advanced mare age impairs the ability of in vitro‐matured oocytes to correctly align chromosomes on the metaphase plate
Source: Equine Vet J. 2018 Aug 9;51(2):252–7. doi: 10.1111/evj.12995 (PMC6585749; doi:10.1111/evj.12995)
Supplement: Supplementary file 1 — Summary in Portuguese. [file EVJ-51-252-s007.docx]

EVJ-GA-18-085.R1

A idade avançada da égua prejudica a capacidade dos oócitos maturados in vitro de alinharem corretamente os cromossomos na placa metafásica

M. Rizzo^a,b,d^, K.D. Ducheyne ^a,c,d,^*, C. Deelen^a^, M. Beitsma^a^, S. Cristarella^b^, M. Quartuccio^b^, T.A.E. Stout^a^, M. de Ruijter-Villani^a^

**Palavras-chaves:** oócitos, envelhecimento materno, morfologia do fuso, desalinhamento cromossomal, cavalo

**Resumo**

**Introdução:** A idade avançada da égua está associada com a redução da fertilidade e o risco aumentado de perda gestacional precoce. A qualidade comprometida do oócito é provavelmente o principal motivo para a redução da fertilidade, todavia os defeitos que predispõem à morte embrionária são desconhecidos. Nas mulheres, a idade avançada predispõe aos erros de separação cromossomal durante a meiose, os quais levam à aneuploidia embrionária e ao risco elevado de aborto espontâneo.

**Objetivos:** Avaliar o efeito da idade avançada da égua no alinhamento cromossomal e na morfologia do fuso meiótico em oócitos maturados *in vitro* (MIV).

**Delineamento Experimental:** Análise morfológica e morfométrica.

**Métodos:** Para investigar as diferenças da organização do fuso e do alinhamento cromossomal entre éguas jovens e velhas, os oócitos coletados de éguas abatidas foram divididos em dois grupos, dependendo da idade da égua (jovem, ≤ 14 anos; velha, ≥16 anos), maturados *in vitro* e corados para visualizar a cromatina e a tubulina alfa. A morfologia do fuso, morfometria e (des)alinhamento cromossomal foram avaliados por microscopia confocal e análise de imagem 3D.

**Resultados:** Os oócitos de éguas velhas apresentaram maior incidência de desalinhamento cromossomal (47.4 % vs. 4.5 %; P < 0.001) e placa metafásica mais espessa (média ± desvio padrão; 5.8 ± 1.0 µm vs. 4.9 ± 0.9 µm; P = 0.04) do que os oócitos de éguas jovens. Embora não tenham sido detectadas quaisquer diferenças na morfologia do fuso entre éguas jovens e velhas, maior comprimento do eixo principal do fuso foi associado com o desalinhamento cromossomal (média ± desvio padrão; 25.3 ± 6.1 µm vs. 20.8 ± 3.3 µm; P = 0.01) independentemente da idade.

**Principais Limitações:** Os oócitos foram maturados *in vitro* e podem não refletir exatamente o desalinhamento cromossomal *in vivo*.

**Conclusão:** A idade avançada da égua predispõe ao desalinhamento cromossomal no fuso da metáfase II de oócitos maturados *in vitro*. A capacidade comprometida de alinhar corretamente os cromossomos provavelmente predispõe à aneuploidia nos embriões resultantes, contribuindo assim para a redução da fertilidade relacionada à idade e ao aumento da incidência de perda embrionária precoce.
